# Afferent nerve activity in a mouse model increases with faster bladder filling rates in vitro, but voiding behavior remains unaltered in vivo

**DOI:** 10.1152/ajpregu.00156.2022

**Published:** 2022-09-19

**Authors:** Thomas J. Heppner, Grant W. Hennig, Mark T. Nelson, Gerald M. Herrera

**Affiliations:** ^1^Department of Pharmacology, University of Vermont, Burlington, Vermont; ^2^Institute of Cardiovascular Sciences, University of Manchester, Manchester, United Kingdom

**Keywords:** incontinence, micturition, overactive bladder, polydipsia, polyuria

## Abstract

Storage and voiding functions in urinary bladder are well-known, yet fundamental physiological events coordinating these behaviors remain elusive. We sought to understand how voiding function is influenced by the rate at which the bladder fills. We hypothesized that faster filling rates would increase afferent sensory activity and increase micturition rate. In vivo, this would mean animals experiencing faster bladder filling would void more frequently with smaller void volumes. To test this hypothesis, we measured afferent nerve activity during different filling rates using an ex vivo mouse bladder preparation and assessed voiding frequency in normally behaving mice noninvasively (UroVoid). Bladder afferent nerve activity depended on the filling rate, with faster filling increasing afferent nerve activity at a given volume. Voiding behavior in vivo was measured in UroVoid cages. Male and female mice were given access to tap water or, to induce faster bladder filling rates, water containing 5% sucrose. Fluid intake increased dramatically in mice consuming 5% sucrose. As expected, micturition frequency was elevated in the sucrose group. However, even with the greatly increased rate of urine production, void volumes were unchanged in both genders. Although faster filling rates generated higher afferent nerve rates ex vivo, this did not translate into more frequent, smaller-volume voids in vivo. This suggests afferent nerve activity is only one factor contributing to the switch from bladder filling to micturition. Together with afferent nerve activity, higher centers in the central nervous system and the state of arousal are likely critical to coordinating the micturition reflex.

## INTRODUCTION

The urinary bladder is a hollow organ that functions as a reservoir to store urine but can switch to generate considerable pressures during voiding of urine. Storing urine during filling and emptying at the appropriate time involves a coordinated autonomic reflex that is under direct control of the central nervous system (CNS; 1). The filling phase is marked by an increase in sensory outflow from the bladder that gradually increases as the bladder fills. This process likely reflects increases in the pressure or distension of the bladder wall that occur during filling.

Sensory information is conducted to the CNS primarily through pelvic and hypogastric nerves. Afferent nerve fibers are composed of myelinated Aδ-fibers and unmyelinated C-fibers. Aδ-fibers are the main contributors to the sensing of bladder fullness, as they respond to bladder wall distension and contraction. C-fibers are normally silent under physiological conditions and respond primarily to noxious stimuli (2).

Ex vivo bladder preparations using multifiber recordings measure afferent nerve activity emanating from the urinary bladder. As the bladder fills and the bladder wall distends, sensory nerve activity increases (3–8). During filling, the bladder also exhibits localized transient contractions, referred to as micromotions, spontaneous phasic contractions, or transient pressure events. These localized events, entailing movements of the bladder wall and changes in intravesical pressure, are nonvoiding contractions that can be measured urodynamically. The function of these events is unknown, but they may be important in regulating bladder tone and conditioning the bladder to accommodate increasing volumes during the filling phase (9–13). Notably, these transient contractions evoke large bursts of afferent nerve activity (5, 14). Sensory outflow from the bladder likely conveys important bladder fullness-related information to the CNS (2, 13, 15). Our group previously found that the rate of bladder wall distortion during transient contractions is a key determinant of afferent nerve activity. Specifically, we found that afferent nerve activity is activated during the rising phase of the transient contraction, such that the faster the rate of rise of the transient contraction, the greater the amount of afferent nerve activity (5). Extending this observation, in the present study, we hypothesized that the increased bladder wall distortion associated with a faster filling rate would also generate a greater amount of afferent nerve activity. In support of this idea, Daly et al. (3) previously reported that afferent nerve activity is higher with higher filling rates. However, their study used very high nonphysiological filling rates (50 and 200 µL/min) and pressures (40–50 mmHg).

Very little is known about the effect of bladder filling rate on voiding function in vivo. Filling rates used in cystometry are generally several fold higher than physiological rates of bladder filling, a fact that may complicate the ability to draw meaningful conclusions about physiological bladder function based on measurements obtained in an experimental setting. Indeed, in clinical and preclinical urodynamics studies, there is some evidence that the rate at which the bladder is filled adversely affects measured urodynamics parameters (16–19).

In the present study, we wanted to gain a better understanding of whether bladder afferent nerve activity depends on the rate at which the bladder is filled, and if so, whether bladder filling rate fundamentally alters voiding behavior in vivo. We tested a range of filling rates estimated to span the range from physiological to supraphysiological (3–30 µL/min). Based on our earlier work (5, 8), we hypothesized that bladder afferent nerve activity would increase to higher levels when faster filling rates were used. This would affect voiding function in vivo, such that animals experiencing faster bladder filling rates would not only void more frequently but also would void smaller volumes, reflecting the fact that the urge to void would be reached at lower bladder volumes owing to the higher level of corresponding afferent nerve activity.

We explored these hypotheses by first examining afferent nerve activity as a function of various bladder filling rates using an ex vivo bladder-filling model. We then tested our hypothesis in vivo using a mouse model of sucrose-induced polydipsia. Mice ingest many more times the volume of liquid when they consume sucrose solution than when they ingest tap water (20, 21). Thus, the a priori expectation was that this increase in liquid consumption should also be associated with increased urine production and hence faster bladder filling rates.

## MATERIALS AND METHODS

### Animals

Male and female C57Bl/6 mice (3–4 mo of age; Jackson Laboratory, Bar Harbor, ME) were used for all studies. Most experiments were conducted using male mice. One voiding assay, described in the section titled *Noninvasive Voiding Frequency Measurements: 72-h Test*, was conducted in both male and female mice to determine if there are any gender differences. All animal usage was reviewed and approved by the Institutional Animal Care and Use Committee at the University of Vermont.

### Ex Vivo Bladder Preparation

Mice were euthanized by intraperitoneal injection of pentobarbital sodium (150 mg/kg) followed by decapitation. The urinary bladder, urethra, ureters, postganglionic nerves, major pelvic ganglia, and pelvic nerves were carefully removed and placed in a cold HEPES physiological saline solution (PSS) consisting of 134 mM NaCl, 6 mM KCl, 1 mM MgCl_2_, 2 mM CaCl_2_, 10 mM HEPES, and 7 mM glucose (pH 7.4). Both ureters were tied with 5/0 silk suture close to the bladder wall, and pelvic nerves were carefully cleaned of connective tissue. The preparation was then placed in a recording chamber and superfused with bicarbonate PSS consisting of 118.5 mM NaCl, 4.6 mM KCl, 1.2 mM KH_2_PO_4_, 1.2 mM MgCl_2_, 2 mM CaCl_2_, 24 mM NaHCO_3_, and 7 mM glucose. pH was maintained at 7.4 by bubbling the solution with 20% O_2_-5% CO_2_. The bladder was then cannulated via the urethra and attached to a variable-speed syringe pump to allow for continuous infusion of bicarbonate PSS into the bladder. All experiments were performed at 37°C. Urinary bladders were continuously filled at 0.2, 0.6, or 1.8 mL/h (corresponding to 3.3, 10, and 30 µL/min, respectively) to a pressure of 25 mmHg. Once this pressure was reached, the bladder was emptied through the urethral cannula. The maximum bladder pressure chosen was based on cystometric recordings from normal C57Bl/6 mice, which indicated that voiding threshold was reached at ∼11 mmHg and peak contraction pressures occurred at ∼26 mmHg (22). Bladder pressure was measured using a pressure transducer placed in the infusion line adjacent to the bladder and connected to a Living Systems Pressure Servo Controller (model PS-200; Living Systems Instrumentation, St. Albans, VT). One of the pelvic nerves was then placed in a recording suction electrode for recording of afferent nerve activity from the urinary bladder.

### Electrophysiology

One of the pelvic nerves was positioned in a glass fire-polished tip (tip opening, ∼100 μm) of a suction electrode using negative pressure. Action potentials recorded from the pelvic nerve were collected using a Neurolog headstage (NL100AKS; Digitimer, Hertfordshire, UK), amplified with an AC preamplifier (NL104; Digitimer), and band-pass filtered at 200–4,000 Hz (NL125/NL126; Digitimer) to remove noise. Data were collected and stored using a Power 401 analog-to-digital interface and Spike 2 software (Cambridge Electronic Design, Cambridge, UK). Pressure was acquired at a rate of 100 Hz, and afferent activity was acquired at a rate of 25,000 Hz. The threshold for detecting afferent nerve activity was set at twice the root mean square of the recorded signal in the absence of action potentials, a level well-above signal noise. Bladder pressure and afferent nerve activity were recorded simultaneously and analyzed offline using Spike 2 software (Cambridge Electronic Design). Action potential frequency was calculated from detected action potentials. “Afferent activity” was defined as the number of action potentials per second (Hz).

### Noninvasive Voiding Frequency Measurements: 48-h Test

Normal spontaneous urinary voiding patterns were studied in mice during a 48-h test session in a commercially available specialized home-cage system (UroVoid, Med Associates, Inc.). Mice were placed individually in a home cage measuring 18.8 cm wide by 13.2 cm deep by 12.7 cm tall. The cage floor was constructed of stainless steel grid rods 1.6 mm in diameter spaced 6.8 mm apart, which allowed feces and urine to pass freely beneath the cage. Urine and feces were separated using a fine stainless steel wire filter placed below the cage floor. Fecal pellets remained trapped on the wire filter screen, whereas urine passed below the screen, where it landed on an aluminum catch pan situated on a weighing scale. A data-acquisition computer running UroVoid Data Acquisition Software (Med Associates, Inc.) collected data from the weighing scale at a resolution of 0.001 g and a rate of four samples per second. Data were analyzed offline using UroVoid Analysis Software (Med Associates, Inc.). The following void detection parameters were used: trigger threshold, 0.001–0.003 g; void threshold, 0.01–0.02 g; void window, 100 s. During the voiding frequency study, mice had free access to food and water. Food was made available in powdered form, prepared by grinding stock food pellets (ProLab Rat/Mouse/Hamster 3000; LabDiet) to a coarse powder using either a mortar and pestle or a small kitchen blending appliance and then passing the coarse powder through a sieve (No. 12 ASTM E11 Standard Test Sieve; W.S. Tyler Industrial Group). Using powdered feed prepared in this manner minimizes hoarding and caching of food and fouling of urine samples by food debris while the mice are housed in the UroVoid apparatus. One group of mice (*n* = 7) had access to normal tap water. The second group of mice (*n* = 8) had access to tap water containing 5% wt/vol sucrose, prepared fresh at the start of each test session. Test sessions lasted 48 h, with food and water consumption checked every 24 h, and body weight measured at the start and end of the session. The light:dark schedule was lights on at 6 AM and lights off at 6 PM. Studies were started approximately between 8 AM and 9 AM.

### Noninvasive Voiding Frequency Measurements: 72-h Test

In the 48-h UroVoid study, separate groups of mice ingested sucrose or tap water during voiding measurements. To examine voiding function in the face of a wide variety of bladder filling rates within the same animal, we used a 72-h modification of this study in which mice (*n* = 8 males and *n* = 8 females) in the UroVoid apparatus were supplied tap water for the initial 24 h, 5% sucrose during the second 24 h, and tap water for the final 24 h. In the male cohort, body weight, and food and water intake were measured daily. For the female mice, food and water intake were measured daily, but body weight was recorded only at the start and completion of the study.

### Statistics and Data Analysis

All summary data are expressed as means ± SE. Statistical methods used are stated in figure legends and table captions, or in the text, as appropriate. A *P* value < 0.05 was used as the criterion for rejecting the null hypothesis in all cases.

## RESULTS

### Effects of Bladder Filling Rate on Afferent Nerve Activity Ex Vivo

To assess the effect of filling rate on afferent nerve activity, we subjected isolated canulated urinary bladders to filling at three different rates, 0.2, 0.6, and 1.8 mL/h (3.3, 10, and 30 µL/min, respectively), and monitored sensory nerve frequency using a suction electrode. These filling rates were specifically chosen to cover the normal physiological range (0.2 and 0.6 mL/h; 3.3 and 10 µL/min) and a high-end physiological rate (1.8 mL/h; 30 µL/min).

**Figure 1.**
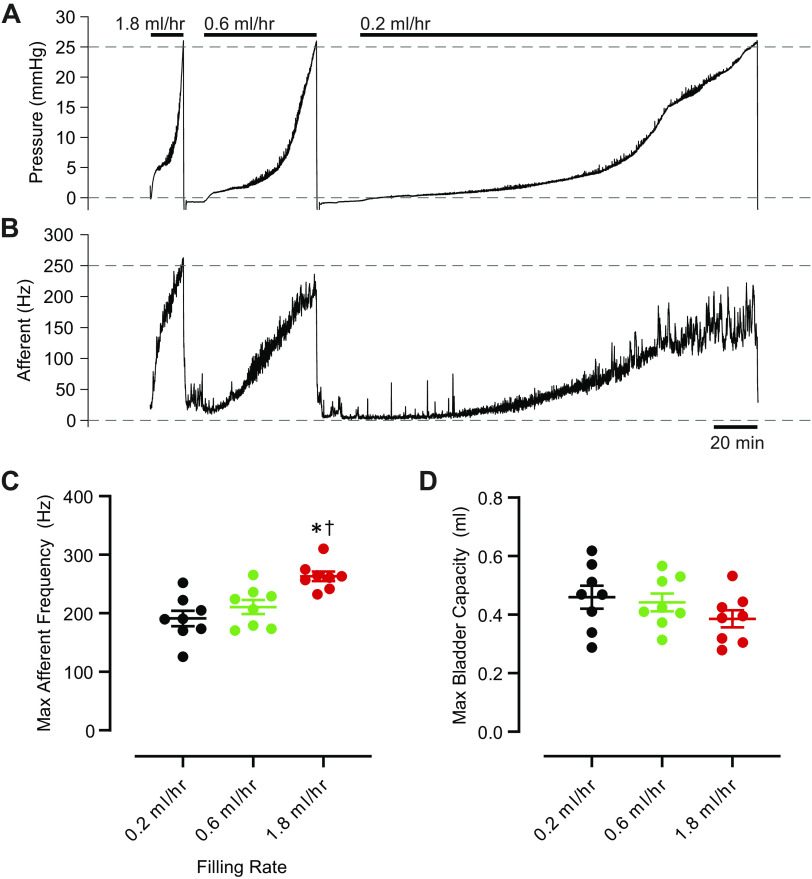
Afferent nerve activity increases with faster bladder filling rates ex vivo. Original recordings of bladder pressure (*A*) and afferent nerve frequency (*B*) in a representative, isolated, cannulated mouse urinary bladder subjected to filling with saline at rates of 0.2, 0.6, and 1.8 mL/h, in descending fashion. At each rate, bladder was filled until intravesical pressure reached 25 mmHg, at which time the filling was stopped and the bladder was emptied. *C*: maximum afferent nerve frequency reached during filling at each of three filling rates tested. Points represent individual bladder measurements (*n* = 8), and horizontal lines indicate means ± SE. *P < 0.05 vs. 0.2 mL/h. †*P* < 0.05 vs. 0.6 mL/h. *D*: volume reached at the end of each filling (Max Bladder Capacity). Points represent individual bladder measurements (*n* = 8), and horizontal lines indicate means ± SE. There were no significant differences. For *C* and *D*, a one-way repeated measures ANOVA followed by Tukey’s test for multiple comparisons was used.

As previously observed (3–8), afferent nerve frequency increased during ex vivo bladder filling (Fig. 1). When bladder pressure reached 25 mmHg, filling was stopped, bladders were emptied, and another filling cycle was commenced. We found that faster filling rates were associated with higher maximum afferent nerve frequencies (Fig. 1, *A*–*C*). Bladder capacity, defined as the volume of infused saline at the end of the filling cycle, was not different across the various filling rates (Fig. 1*D*).

To ascertain whether the effects of bladder filling rate on maximum afferent nerve frequency depended on the initial conditions, we conducted experiments in which the bladder filling rate changes were applied in an ascending fashion, from 0.2 mL/h to 0.6 mL/h to 1.8 mL/h (*n* = 4 bladders), and another set of experiments (*n* = 4 bladders) in which the filling rate changes were applied in a descending fashion from 1.8 mL/h to 0.6 mL/h to 0.2 mL/h. We found no significant difference regardless of whether filling started from a high rate and changed to low or started from a low rate and changed to high. Specifically, when filling rate was varied in an ascending fashion, the maximum afferent nerve frequency recorded increased from 103.7 ± 46.0 Hz to 132.2 ± 54.3 Hz and then to 167.7 ± 62.6 Hz at 0.2, 0.6, and 1.8 mL/h, respectively. When the filling rates were changed in descending fashion from 1.8 to 0.6 to 0.2 mL/h, the corresponding maximum afferent nerve frequency recorded decreased from 222.6 ± 21.9 Hz to 169.6 ± 18.1 Hz to 127.9 ± 15.4 Hz. The maximum afferent nerve frequency recorded at any given filling rate was not significantly different between ascending- and descending-fill groups (two-way repeated measures ANOVA with Sidak test for multiple comparisons). This observation suggests that afferent nerve frequency depends directly on the filling rate used and is not related to residual effects or other technical factors, such as rundown of the preparation over time or loss of suction at the suction electrode.

**Figure 2.**
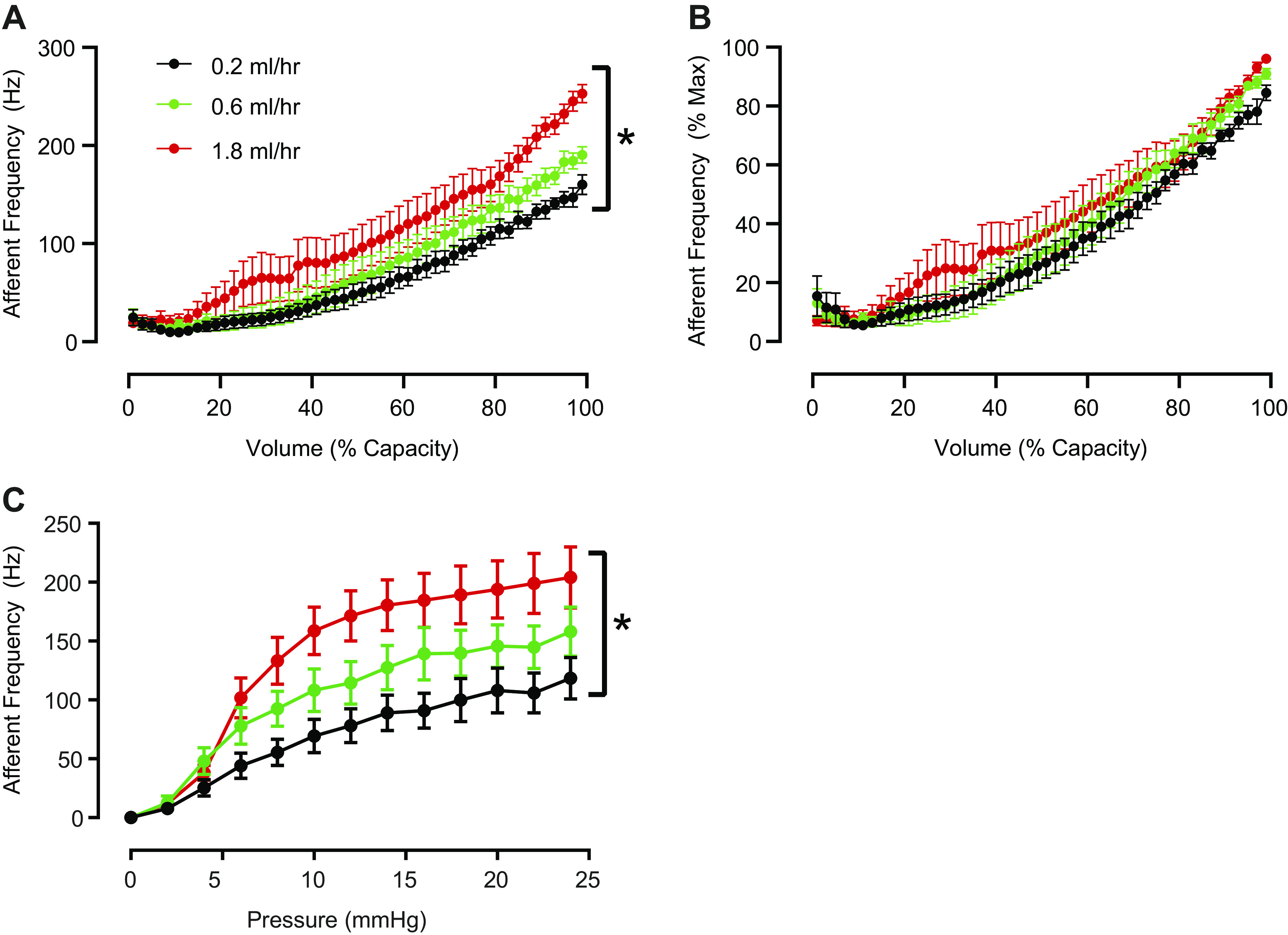
Maximal afferent nerve frequency reached during filling increased at faster filling rates, but the sensitivity of afferent nerves did not change. *A*: absolute afferent nerve activity plotted against normalized bladder capacity (see text for description) at each filling rate (*n* = 8 bladders). *P < 0.05 for all filling rates. *B*: normalized afferent nerve activity (%Max) expressed as a function of normalized bladder capacity at each filling rate (*n* = 8 bladders). Filling rate did not significantly affect normalized nerve activity relative to the fractional filling state. *C*: absolute afferent nerve activity plotted against bladder pressure in 2 mmHg bins. *P < 0.05 for all filling rates. Data were analyzed using a two-way repeated measures ANOVA followed by Tukey’s test for multiple comparisons.

Despite the absence of differences in bladder capacity (Fig. 1*D*), there was noticeable variability in bladder capacity among the individual bladders tested. To account for this interpreparation variability, we normalized filling volumes such that the volume reached at the end of a fill (25 mmHg indicating full bladder capacity) was defined as 100% and plotted afferent nerve frequencies against normalized bladder capacity (Fig. 2*A*). We found that faster filling rates were associated with larger overall increases in afferent nerve frequency (Fig. 2*A*). We next normalized afferent nerve frequencies, such that the maximum frequency achieved during a filling cycle was defined as 100%. When normalized nerve frequencies were plotted against normalized bladder capacities, there were no longer any differences among the various filling rates tested (Fig. 2*B*). We also examined the relationship between afferent nerve frequency and bladder pressure, and afferent nerve activity increased to a greater extent with faster bladder filling rates across a wide range of bladder pressures (Fig. 2*C*). Thus, it appears that the maximum afferent nerve frequency increases with faster filling, but the rate at which the nerve activity increases during filling does not change. This finding suggests that faster filling rates may be associated with recruitment of additional nerve units, rather than changes in individual nerve unit firing frequencies.

### Effects of Excess Fluid Consumption on Voiding Behavior in Mice

**Figure 3.**
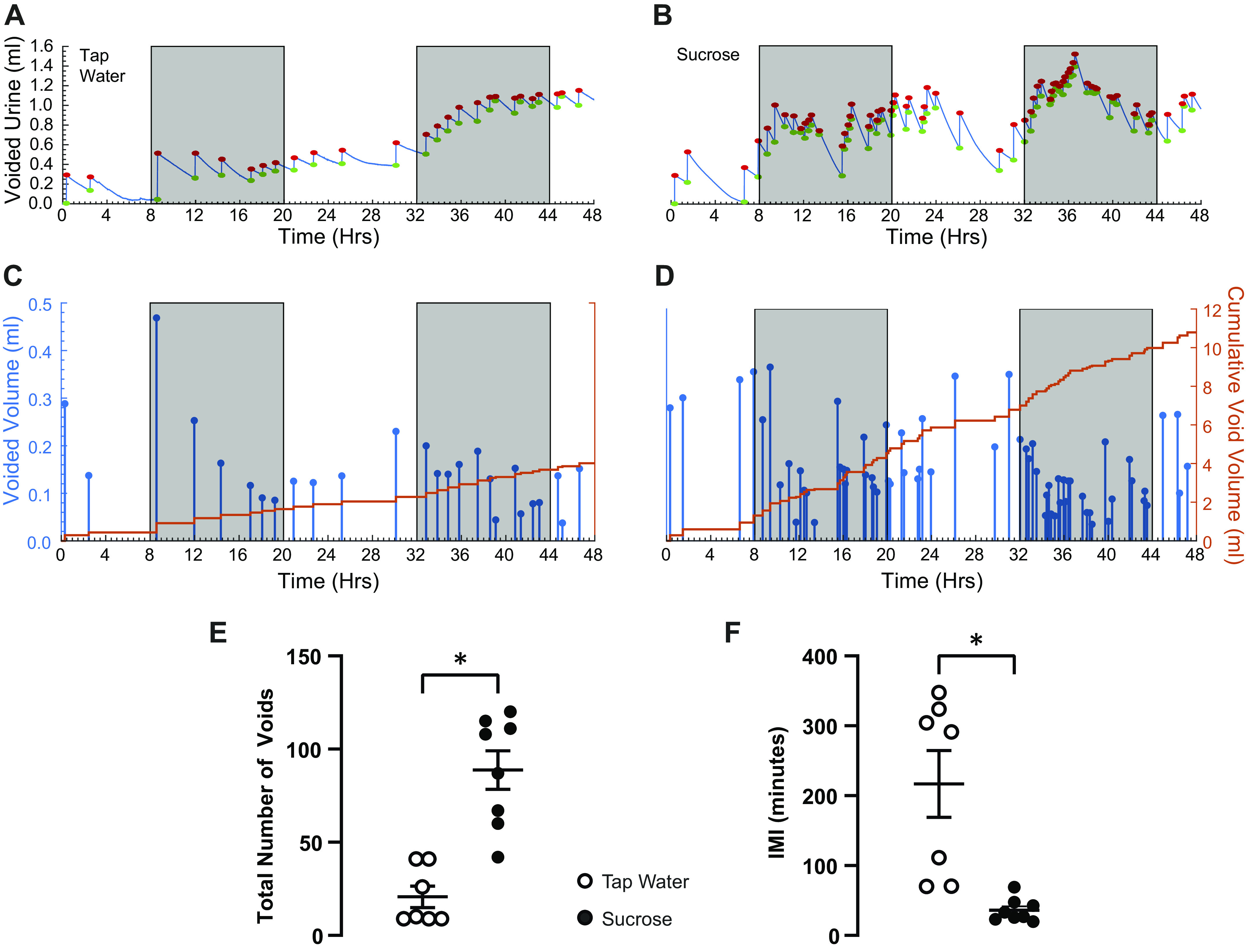
Original voiding micturograms from mice ingesting tap water (*A* and *C*) and 5% sucrose (*B* and *D*). *A* and *B*: raw data from the analytical balance that weighed each void event. Baseline (green dots) and peak (red dot) for each void is indicated. Shaded rectangles indicate the dark phase of the light:dark cycle. *C* and *D*: void volumes for every void extracted from raw data, plotted as individual void volumes (blue dots/lines, left *Y*-axis), or as a cumulative record with each void volume added to the prior void volume (orange lines, right *Y*-axis). Shaded rectangles indicate the dark phase of the light:dark cycle. *E*: total number of voids during the 48-h voiding study observed in mice consuming tap water (*n* = 7 mice) or 5% sucrose (*n* = 8 mice). *F*: average intermicturition interval (IMI) for all void events from each animal recorded during the 48-h voiding study. *E* and *F*, individual subject data are plotted as circles, and horizontal lines indicate means ± SE. *P < 0.05 vs. tap water. An unpaired Mann–Whitney test on ranks was used for statistical analysis.

**Figure 4.**
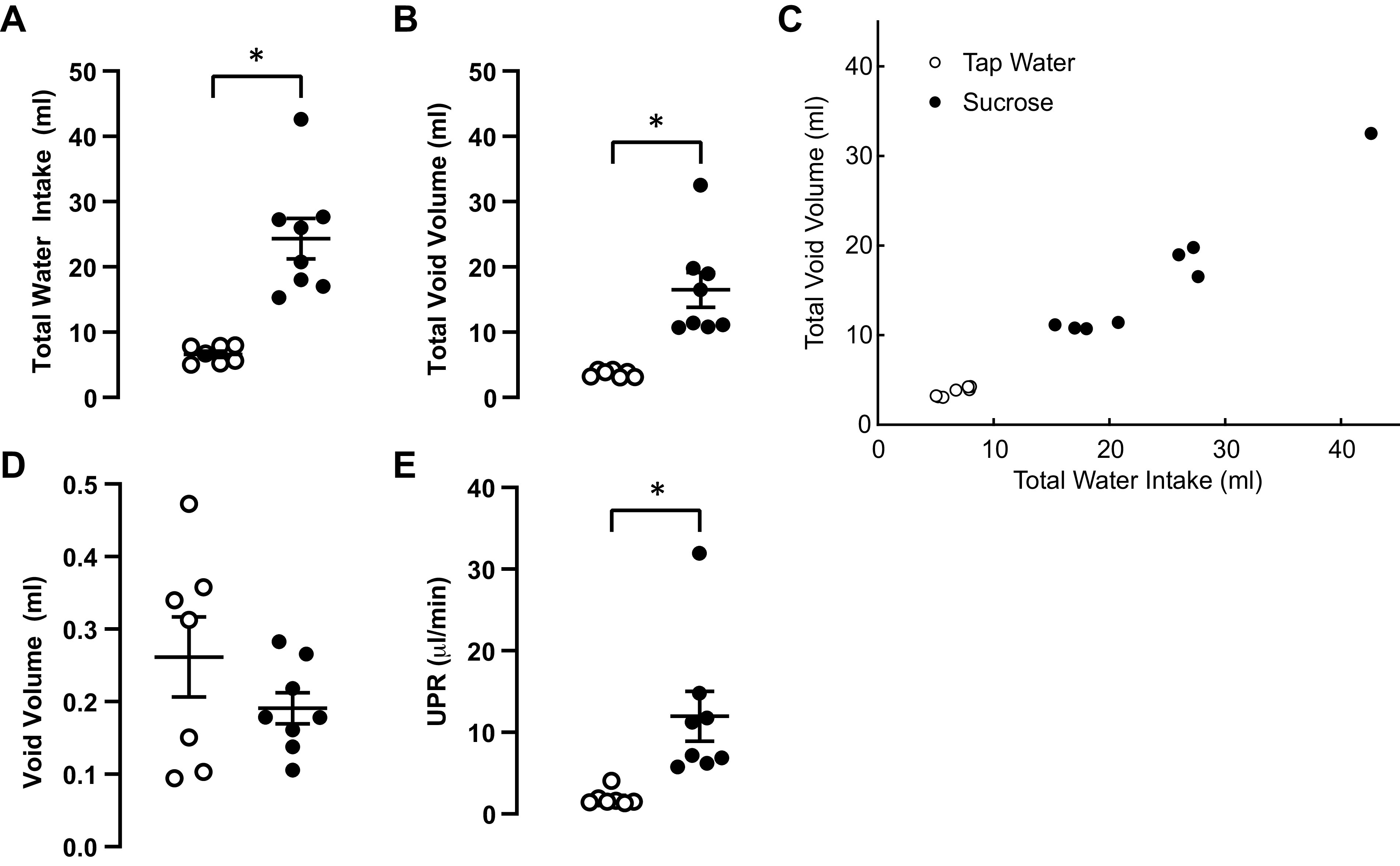
*A*: total water intake during the 48-h voiding study for mice consuming tap water (open circles, *n* = 7 mice) or 5% sucrose (closed circles, *n* = 8 mice). *B*: total void volume during the 48-h voiding study. *C*: correlation plot showing total void volume plotted against total water intake for all individual subjects. *D*: average void volume obtained by averaging the volume of all individual void events detected in each subject. *E*: average urine production rate (UPR) obtained by averaging all individual void events from each subject. In all panels, individual subject data are plotted as circles, and horizontal lines indicate means ± SE. *P < 0.05 vs. tap water. An unpaired Mann–Whitney test on ranks was used for all statistical analyses.

Because maximal afferent nerve activity increases with faster filling rates in the isolated mouse urinary bladder, we hypothesized that voiding behavior would be altered in vivo when mice experience faster than normal bladder filling rates. Specifically, we anticipated that average void volumes would be reduced when mice experience fast bladder filling rates since we would expect sensory nerve activity to reach high enough rates to trigger the urge to void at lower bladder volumes. To test this hypothesis, we used a common model of polydipsia in which mice are provided tap water containing sucrose. Because we wanted to minimize disruptions in energy metabolism, we opted for a low level of sucrose (5%) for an acute period (48 h). Mice provided 5% sucrose in their drinking water and consumed approximately fourfold higher volumes of liquid during this 48-h test, with no change in food intake (Table 1). Voided urine volume in mice drinking sucrose was elevated nearly fivefold compared with those consuming tap water (Figs. 3 and 4).

**Table 1.** Daily body weight, liquid, and food intake for mice ingesting tap water or 5% sucrose during the 48-h voiding study

	Tap Water (*n* = 7)	5% Sucrose (*n* = 8)
Initial body weight	26.84 ± 2.14 g	25.05 ± 0.87 g
Final body weight	25.59 ± 1.81 g*	24.49 ± 0.83 g
Change in body weight	−4.3 ± 0.9 %	−2.2 ± 0.9 %
Food consumption *day 1*	2.30 ± 0.21 g	2.83 ± 0.33 g
Food consumption *day 2*	3.06 ± 0.29 g	3.07 ± 0.30 g
Total food consumption	5.35 ± 0.42 g	5.90 ± 0.54 g
Water consumption *day 1*	2.87 ± 0.54 mL	11.80 ± 1.00 mL†
Water consumption *day 2*	3.81 ± 0.37 mL	12.52 ± 2.75 mL†
Total water consumption	6.61 ± 0.50 mL	24.32 ± 3.12 mL†

Results are means ± SE. *n*, number of mice in each group. *P < 0.05 vs. initial body weight. †*P* < 0.05 vs. tap water, two-way repeated measures ANOVA with Sidak test for multiple comparisons.

To explore the relationship between bladder filling rate and voiding function in more detail, we examined how the time between each void (intermicturition interval) and void volume related to one another. Figure 3, *A* and *B*, shows the raw outputs from a 48-h voiding study session. Each time the mouse voids urine, the drop of urine lands on a weighing scale and generates an upward deflection. Then the urine evaporates, and the signal starts to decay. Voided urine volumes are obtained by measuring the weight detected by the weighing scale immediately before a void event and subtracting it from the weight detected 100 s after the void is initiated. This time period, which is user-selectable, was chosen based on preliminary tests showing that this time frame was long enough to capture any dribbling that occurred but short enough to minimize measurement artifacts caused by urine evaporation.

**Figure 5.**
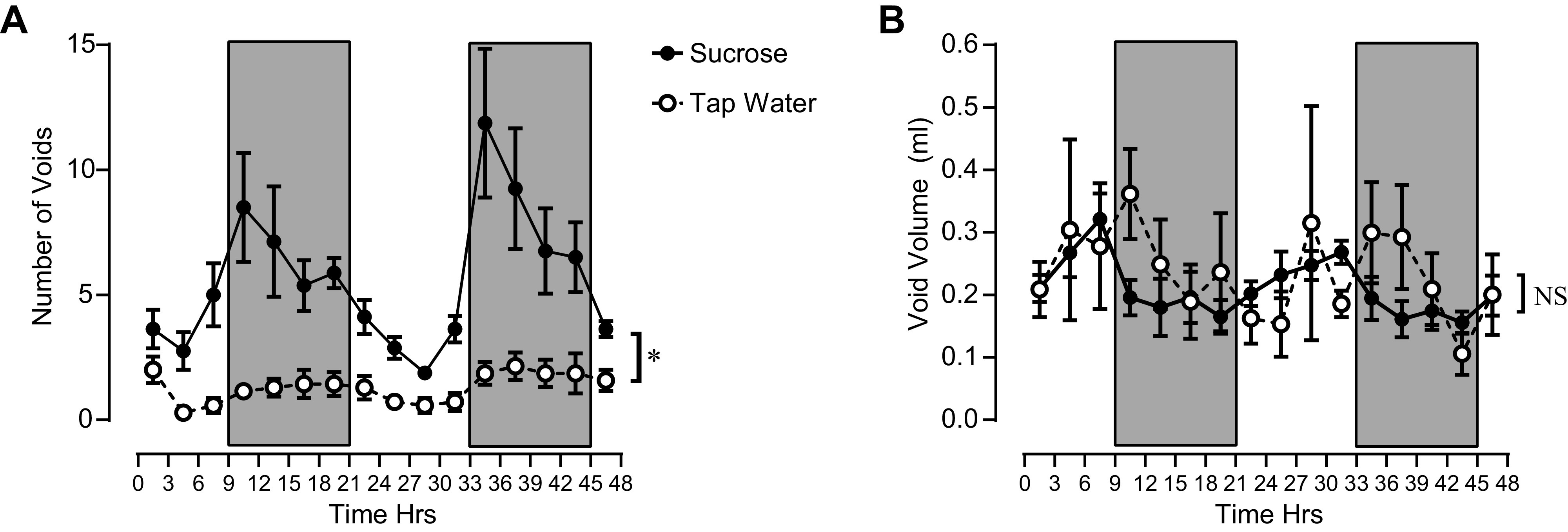
*A*: number of voids detected during 3-h time bins in mice consuming tap water (open circles, *n* = 7 mice) or 5% sucrose (closed circles, *n* = 8 mice). *B*: average void volumes recorded during 3-h time bins in mice consuming tap water or 5% sucrose. Shaded rectangles indicate dark portion of the light:dark cycle. *P < 0.05 between the tap water and sucrose groups. NS, no significant difference between tap water and sucrose groups. A two-way repeated measures ANOVA with Greenhouse–Geisser correction for unequal variances was used for all statistical comparisons.

**Figure 6.**
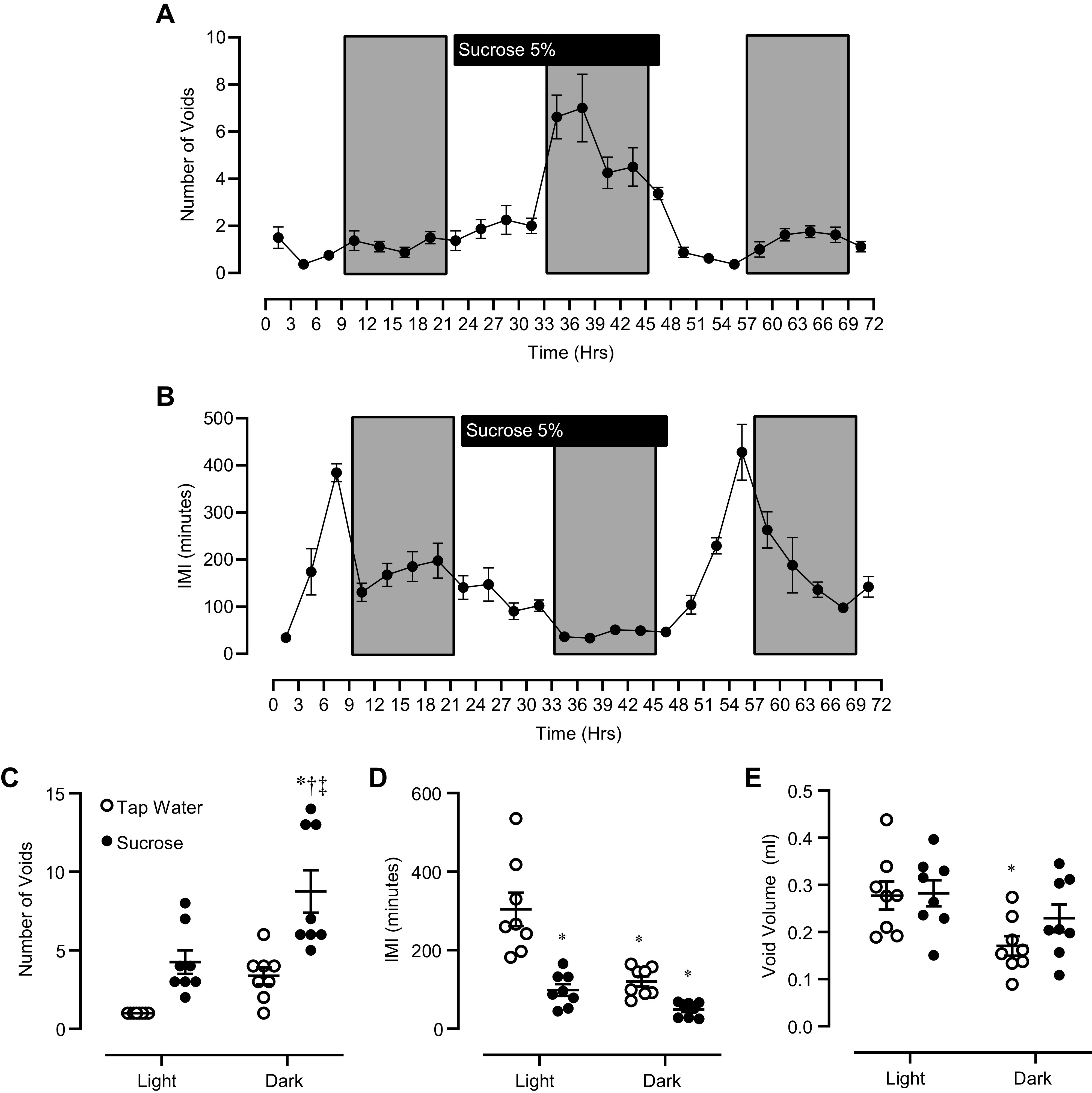
Seventy-two hour voiding behavior in male mice (*n* = 8 mice). *A*: number of voids measured in 3-h time bins during the 72-h voiding study in which mice had access to tap water for the first and third 24-h periods and 5% sucrose water during the second 24-h period. Shaded rectangles indicate the dark portion of the light:dark cycle. *B*: average intermicturition interval (IMI) detected in 3-h time bins during the 72-h voiding study. *C*: average number of voids detected in the light and dark phases of the light:dark cycle in mice drinking tap water (open circles) and 5% sucrose (closed circles). *D*: average IMI detected in light and dark phases of the light:dark cycle. *E*: average void volumes measured in light and dark phases of the light:dark cycle. For *C–E*, voiding behavior was quantified during the final 6 h of light/dark cycles. Individual subject data are plotted as circles, and horizontal lines are means ± SE. *P < 0.05 vs. tap water light cycle. †*P* < 0.05 vs. sucrose light cycle. ‡*P* < 0.05 vs. tap water dark cycle. A two-way repeated measures ANOVA with Tukey’s test for multiple comparisons was used for all statistical analyses.

As expected (23), voiding behavior in mice followed a striking nocturnal pattern (Figs. 3, 5, and 6). During the dark phase of the light:dark period, mice voided 3–4-times more often than during the light phase. Void volumes tended to be slightly smaller during the dark phase, and voids were spaced closer together (i.e., intermicturition intervals decreased). In mice drinking 5% sucrose, this nocturnal pattern persisted, even though, during the light phase, they voided similarly to mice drinking tap water during the dark phase (Fig. 3).

Mice drinking 5% sucrose consumed more water and voided more often than did mice drinking tap water (Fig. 4). Thus, they were clearly producing urine at a faster rate than mice drinking tap water (Fig. 4*E*). However, because we did not compare voiding within a single subject in these experiments, we could not be sure if the differences in voiding patterns were related to animal-to-animal variations in bladder capacity, the fact that they were making more urine or, more interestingly, whether the rate at which urine fills the bladder influences voiding behavior.

**Figure 7.**
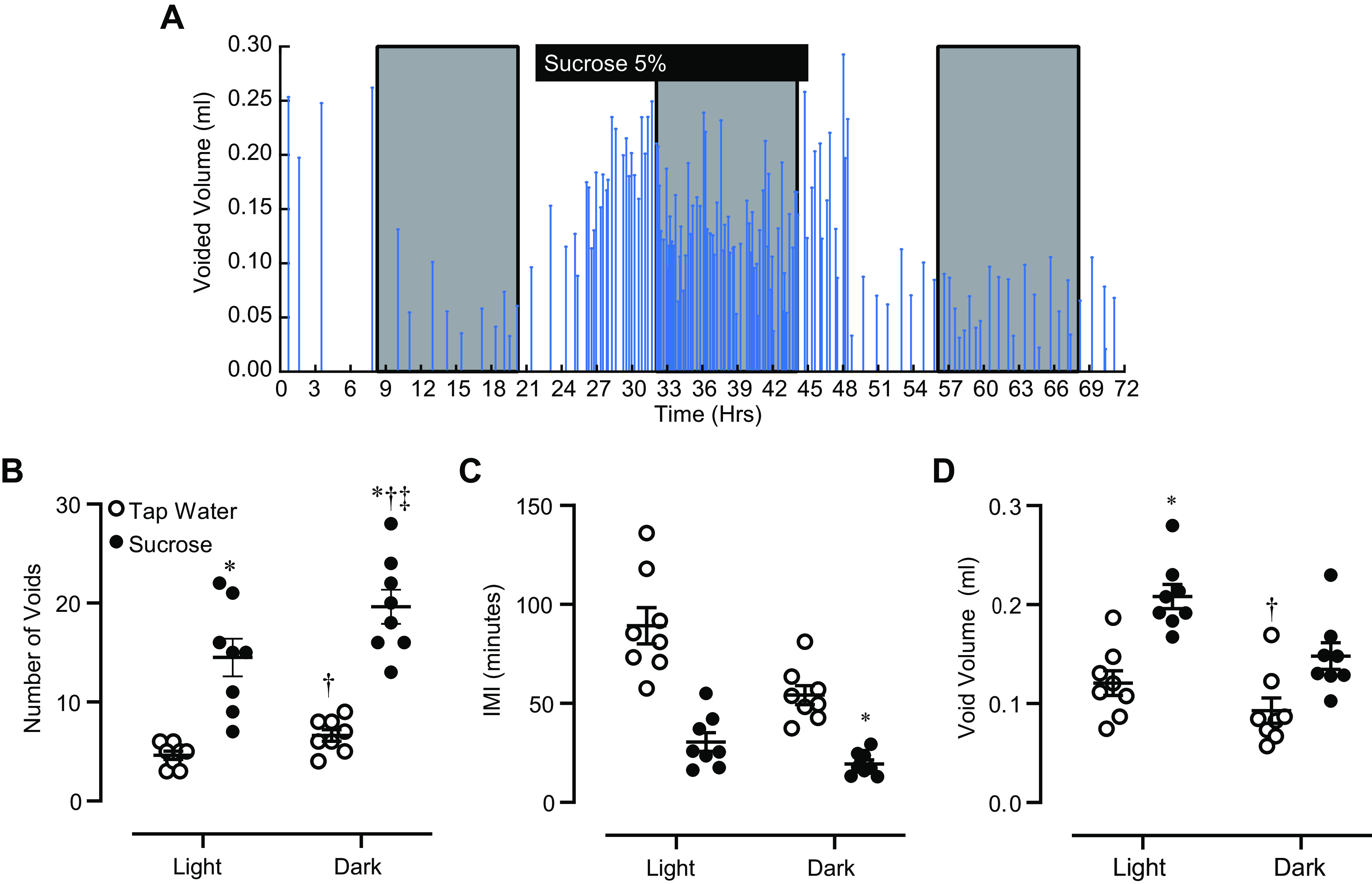
Seventy-two hour voiding behavior in female mice (*n* = 8 mice). *A*: original voiding micturogram obtained from a female mouse during the 72-h voiding study in which mice had access to tap water for the first and third 24-h periods and 5% sucrose water during the second 24-h period. Shaded rectangles indicate the dark portion of the light:dark cycle. *B*: average number of voids detected in the light and dark phases of the light:dark cycle in mice drinking tap water (open circles) and 5% sucrose (closed circles). *C*: average IMI detected in light and dark phases of the light:dark cycle. *D*: average void volumes measured in light and dark phases of the light:dark cycle. For *B–D*, voiding behavior was quantified during the final 6 h of the light:dark cycles. Individual subject data are plotted as circles, and horizontal lines are means ± SE. *P < 0.05 vs. tap water light cycle. †*P* < 0.05 vs. sucrose light cycle. ‡*P* < 0.05 vs. tap water dark cycle. A two-way repeated measures ANOVA with Tukey’s test for multiple comparisons was used for all statistical analyses. IMI, intermicturition interval.

To assess voiding behavior in each subject under a wide range of urine production rates, we modified our experimental procedure to allow us to observe voiding behavior in the same subjects consuming tap water or 5% sucrose solution. We conducted this test in both male and female mice to determine if there are any gender-related differences in voiding behavior when mice experience a wide range of urine production/bladder filling rates. When male mice were given access to 5% sucrose water, they consumed ∼2.5-fold more liquid by volume than when they ingested tap water (Table 2). Similarly, cumulative voided urine volume was greatly increased (3.5- to fourfold) when male mice drank sucrose compared with tap water (Table 2). In female mice, the polydipsia/polyuria was even more pronounced (Table 3). Female mice increased liquid consumption 4.6-fold when ingesting sucrose water, and their total void volume increased sixfold (Table 3). In both male and female mice, voiding activity followed a typical nocturnal pattern, with more frequent voids observed during the dark portion of the light:dark cycle (Fig. 6, *A* and *C*; Fig. 7, *A* and *B*). Because of animal-to-animal variability, the increase in number of voids during the dark cycle relative to light cycle only reached statistical significance during the period when mice consumed a sucrose solution (Figs. 6*C* and 7*B*). In males, the intermicturition intervals were significantly shorter during the dark phase compared with the light phase when mice drank tap water (Fig. 6*D*), whereas in female mice, the difference in intermicturition interval between light and dark cycles did not reach statistical significance (Fig. 7*C*).

**Table 2.** Daily body weight, liquid, and food intake for male mice ingesting tap water, 5% sucrose, and tap water during the 72-h voiding study

	*Day 1*: Tap Water	*Day 2*: 5% Sucrose	*Day 3*: Tap Water
Body weight, g	23.39 ± 0.49	23.19 ± 0.45	22.66 ± 0.34
Liquid intake, mL	4.10 ± 0.29	10.95 ± 1.25†‡	3.66 ± 0.22
Food intake, g	2.50 ± 0.18	2.63 ± 0.19	2.65 ± 0.17
Total number of voids	8 ± 1	30 ± 3†‡	11 ± 1
Total void volume, mL	1.73 ± 0.15	7.27 ± 0.92†‡	2.13 ± 0.15

Initial body weight was 23.81 ± 0.54 g. Results are means ± SE; *n* = 8 mice. †*P* < 0.05 vs. tap water (*day* 1). ‡*P* < 0.05 vs. tap water (*day 3*), two-way repeated measures ANOVA with Tukey test for multiple comparisons.

**Table 3.** Body weight, liquid, and food intake for female mice ingesting tap water, 5% sucrose, and tap water during the 72-h voiding study

	*Day 1*: Tap Water	*Day 2*: 5% Sucrose	*Day 3*: Tap Water
Liquid intake, mL	3.23 ± 0.22	15.12 ± 1.49†‡	3.71 ± 0.12
Food intake, g	3.18 ± 0.13	3.70 ± 0.13	4.05 ± 0.12†
Total number of voids	15 ± 2	59 ± 6†‡	33 ± 4†
Total void volume, mL	1.56 ± 0.14	9.36 ± 1.11†‡	3.77 ± 0.28†

Results are means ± SE; *n* = 8 mice. Starting body weight, *day 1*: 22.21 ± 0.36 g. Ending body weight, *day 3*: 22.30 ± 0.28 g. †*P* < 0.05 vs. tap water (*day 1*). ‡*P* < 0.05 vs. tap water (*day 3*), two-way repeated measures ANOVA with Tukey test for multiple comparisons.

Despite the profound polyuria experienced by both male and female mice that consumed a sucrose solution, their average voided urine volumes did not decrease (Figs. 6*E* and 7*D*). In male mice, there was no difference in average void volumes in either light or dark cycles when mice were consuming tap water or sucrose (Fig. 6*E*). In female mice, void volumes during sucrose consumption were higher than during tap water consumption, and this difference was statistically significant during the light cycle (Fig. 7*C*). Again, we anticipated that voided urine volumes would tend to be smaller when mice ingested sucrose water due to the greatly elevated urine production rate subsequent to increased liquid consumption. The fact that voided urine volumes did not decrease during sucrose-induced polydipsia/polyuria suggests that factors other than afferent nerve activity alone are important in coordinating the micturition reflex in vivo.

It is possible that voiding efficiency may be decreased in mice experiencing fast bladder filling rates, such as during sucrose-induced polydipsia/polyuria. Reduced voiding efficiency may occur if the bladder is unable to completely empty after each void, yielding a post void residual volume. We reasoned that any increase in post-void residual volume would be related to the detrusor being unable to fully empty the bladder, and this would be associated with weakened/slower contraction of the detrusor. Any weakness or slowing of detrusor contraction is measurable in the UroVoid assay by looking at the rate at which urine is expelled during a micturition event (Δ weight of urine detected on scale/Δ time). We denote this measure as void velocity (expelled mL of urine/time).

In male mice, average void velocity was 0.038 ± 0.002 mL/s when mice consumed tap water. Conversely, during sucrose consumption, the average void velocity was 0.037 ± 0.002 mL/s, virtually the same as during tap water consumption (*n* = 8 mice). These figures were obtained by averaging all the void velocities for voids during the last 6 h of the light cycle for each mouse under tap water or sucrose consumption, which are the same voids presented in Fig. 6, *C*–*E* and Fig. 7, *B*–*D*. Similar findings were noted when looking at dark cycle void velocities (0.029 ± 0.003 mL/s for tap water and 0.035 ± 0.003 mL/s for sucrose). Interestingly, in female mice, not only were void velocities not decreased in the presence of sucrose consumption, but they were significantly increased relative to tap water consumption (*P* < 0.05, two-way ANOVA followed by Tukey test for multiple comparisons). Average light cycle void velocity was 0.023 ± 0.001 mL/s during tap water and increased to 0.035 ± 0.001 mL/s during sucrose consumption. Dark cycle void velocities increased from 0.021 ± 0.002 mL/s during tap water consumption to 0.028 ± 0.001 mL/s during sucrose consumption.

## DISCUSSION

### Estimating Urine Production Rate Based on In Vivo Void Volumes and Intermicturition Intervals

Prior work suggests that the maximum rate of afferent nerve discharge from the bladder increases with faster bladder filling rates (3). Daly et al. (3) found that the maximal rate of afferent nerve activity recorded from isolated mouse bladders increased in bladders filled at a rate of 12 mL/h (200 µL/min) relative to those filled at 3 mL/h (50 µL/min). In the present study, our goal was to determine if bladder filling rate impacts the rate of afferent discharge from the bladder, and if so, to assess its physiological effects on voiding function. An early consideration in the design of these studies was the question of appropriate filling rate. From the outset, we realized that bladder filling rates used in some ex vivo experiments, and even mouse urodynamic studies, are likely severalfold higher than those achieved during normal physiology. To determine an appropriate bladder filling rate, we started from the assumption that mice fully void their bladder contents with each void, and then calculated filling rate by dividing the volume of urine voided during an individual micturition event under normal conditions by the intermicturition interval (time from the current event to the preceding void event). Using this approach, we estimated that normal physiological bladder filling rates in mice range from 0.06 to 0.6 mL/h (1–10 µL/min, e.g., Fig. 4*E*); filling rates that are up to two orders of magnitude lower than those used in other studies (i.e., 3–12 mL/h; 50–200 µL/min; 3). Indeed, our traditional urodynamic filling rate of 1.8 mL/h (30 µL/min; 22), which we have also used in previous ex vivo bladder function studies (5), is at the high end of physiological urine production rates, likely only encountered during periods of excessive fluid intake or diuresis. Based on these calculations, we specifically chose ex vivo bladder filling rates of 0.2 and 0.6 mL/h (3.3 and 10 µL/min) as physiological rates and included 1.8 mL/h (30 µL/min) to capture the high end of physiological filling rates in our studies of potential impacts of bladder filling rate on afferent nerve discharge during filling.

### Bladder Filling Rate and Ex Vivo Sensory Nerve Activity

Using bladder filling rates that we expected mice would normally encounter, we observed that faster bladder filling rates were associated with increased maximal rates of afferent nerve activity (Figs. 1 and 2), suggesting that afferent nerves respond to the rate at which the bladder is being filled, not just to the overall level of distension or volume experienced during filling.

Expressing absolute afferent nerve discharge frequency relative to the normalized bladder capacity in each preparation revealed a clear filling rate-dependent increase in maximal afferent nerve discharge rate (Fig. 2*A*). Thus, the maximal rate of afferent nerve discharge does increase the faster the bladder is filled. However, normalization of afferent nerve frequency to the maximal rate achieved during each fill eliminated filling rate-dependent effects on afferent nerve discharge (Fig. 2*B*). Thus, although the maximal rate of afferent nerve discharge does increase with faster bladder filling rates, the relative rate at which afferent discharge increases during filling does not depend on the rate at which the bladder is being filled. One way of explaining these observations is that additional afferent nerves are recruited with faster filling rates, whereas the sensitivity of individual sensory nerves to distention does not change. Recruitment of additional nerve fibers during faster fills would result in greater overall levels of nerve activity, which we observed (Fig. 2*A*). Furthermore, if the sensitivity of sensory nerves to distention is unchanged, the rate at which nerve discharge increases during bladder filling would be unaltered across different filling rates, which we also observed (Fig. 2*B*). Further studies using single-unit recordings will be instrumental in elucidating the specific manner in which sensory nerves respond to distention of the bladder wall during filling.

### Does Bladder Filling Rate Affect Voiding Behavior In Vivo?

Given our finding that the maximal rate of afferent nerve discharge does increase with faster bladder filling rates, we hypothesized that the increase in afferent rate at higher bladder filling rates would lead to more frequent, smaller volume voids in vivo. We tested this hypothesis in a sucrose polydipsia model in which mice dramatically increase their liquid intake and consequently produce more urine at a faster rate, and thus void proportionately more urine. A relatively mild protocol was used in which mice were given access to water containing 5% sucrose for a brief period (24 or 48 h, depending on the test). In this way, we sought to minimize any chronic disruptions of metabolism caused by sucrose ingestion and consequent changes this might have on bladder function. Indeed, we found that fluid intake was greatly increased (2.5- to 4.5-fold) in mice given access to 5% sucrose water compared with mice that ingested tap water (Fig. 4*A*, Tables 1 and 2). We further found that mice consuming more liquid also voided more often (Fig. 3, Fig. 5*A*, and Fig. 6, *A*–*D*). Despite more frequent voiding, individual void volumes remained unaltered in mice that consumed more liquid (Figs. 4*D*, 5*B*, and 6*E*). It is well established that rodent voiding behavior follows a nocturnal pattern (23). To avoid overlooking subtle, context-dependent changes in void volume, we also analyzed void volumes according to time of day and light:dark phases of the 24-h light:dark cycle. These analyses showed that individual void volumes did not differ between mice that consumed tap water or 5% sucrose at any time of day (Figs. 5*B* and 6*E*).

Based on individual void volumes and intermicturition intervals recorded in vivo in this study, we estimate that, with normal water consumption patterns, mice produced urine and experienced bladder filling at rates of ∼1 to 4 µL/min, whereas with ingestion of 5% sucrose water, liquid consumption increased together with increases in urine production rates, typically to ∼12 µL/min but in some cases to as high as 30 µL/min (Fig. 4*E*). Factoring these estimates of in vivo urine production/bladder filling rates into our ex vivo observations showing that bladder filling rates on this same scale cause an increase in overall afferent nerve discharge during bladder filling, we expected to see a decrease in average void volume in mice copiously consuming fluids, reasoning that the urge to void would be reached at lower bladder volumes based on higher sensory nerve output from the bladder. Given that average void volumes in vivo remained unchanged despite the higher bladder filling rates, there must be mechanisms in addition to afferent nerve input that coordinate voiding reflex centrally.

### Gender Differences in Voiding Behavior and the Use of UroVoid as Opposed to Void Spot Assay

Male and female mice exhibit different voiding patterns (24), and social context is a strong parameter that affects voiding behavior differentially based on gender (25). In our study, we examined voiding function over several days in vivo in male and female mice in the absence of social cues and hierarchies. This approach led to some interesting findings. We observed that female mice have smaller apparent bladder capacities than male mice, as determined from average void volumes while drinking normal tap water (Figs. 6*E* and 7*D*). Although tap water consumption was similar in female and male mice (Tables 2 and 3) because they have smaller bladder capacities, female mice voided more frequently than male mice (Figs. 6*C* and 7*B*). This finding contrasts with prior observations using void spot assay where female mice deposited fewer void spots and greater overall urine volume than males (24). This discrepancy highlights a limitation of using void spot assay to determine voiding frequency in mice. With void spot assay, it is impossible to know how many voids occurred, and when those voids occurred. Another limitation of the void spot assay is the typically short duration (e.g., 4–6 h) of the recording. It is difficult to accurately assess normal voiding patterns over such a short period of time. In contrast, with the UroVoid assay, each void can be quantified in temporal sequence, and the test can be conducted across several days, yielding a more extensive assessment of voiding patterns. Another interesting finding in our study was that female mice ingesting sucrose water experienced dramatic polyurea/polydipsia, even more pronounced than in male mice (Tables 2 and 3). Despite the substantial increase in bladder filling rate experienced in female mice consuming sucrose solution, average void volumes did not decrease, but in fact increased significantly (Fig. 7*D*). Thus, in both male and female mice, we were unable to detect a decrease in void volume when mice experienced fast bladder filling rates, as we would have predicted based on our ex vivo observation indicating that faster filling rates lead to increases in afferent nerve activity.

### Does Diminished Voiding Efficiency Contribute to Voiding Function in Sucrose-Induced Polydipsia/Polyuria?

If emptying efficiency were to decrease in any of our conditions, we would expect to see slower voiding velocities. We did not see this happen. Void velocities were either unchanged (in male mice) or increased significantly (in female mice) during sucrose consumption. Thus, we believe that it is unlikely that PVR contributes to decreased voiding efficiency under our experimental conditions.

### Other Processes That May Affect Voiding Behavior In Vivo

Micturition reflects the complex interplay of the autonomic nervous system, CNS, and both involuntary and voluntary smooth muscle activity. Switching from urine storage to elimination depends on brain input, and a key to this synchronization is Barrington’s nucleus, located in the rostral pons of the brainstem. Barrington’s nucleus sends projections to the sacral spinal cord to coordinate detrusor contraction and relax the external urethral sphincter (2, 26, 27). Although Barrington’s nucleus controls micturition, it receives direct input from numerous regions of the cortex and forebrain that may influence the change from bladder filling to micturition. Magnetic resonance imaging studies of the activity of cerebral networks, circuits, and local brain regions in humans during bladder filling following natural ingestion of water suggested that specific brain regions, such as the medial prefrontal cortex, interact with arousal centers to play a role in shifting focus from ongoing activities unrelated to micturition to a focus on voiding in an appropriate environment (28). Thus, arousal state, which was uncontrolled in the current study, is likely a key factor in triggering micturition. This could indicate that drinking sucrose water maintains mice in a more aroused state. Accordingly, future studies should consider reevaluating this question using a model in which liquid intake and urine production rate can be varied while maintaining the same arousal state in all animals. This may require conscious ambulatory urodynamics in which mice are subjected to different bladder filling rates during various phases of the light:dark cycle.

The constancy of void volumes, regardless of filling rates, was surprising and suggests that the voiding reflex may be engaged at a specific volume threshold. This opens the intriguing possibility that it is not only raw afferent activity that initiates the voiding reflex, but additional signaling incorporating wall characteristics at a specific bladder volume. Such signaling could either be derived from the pattern of afferent outflow or integrated in higher brain centers. Indeed, high-frequency bursts of afferent activity associated with transient pressure events at subthreshold volumes do not trigger the voiding reflex, even though the peak frequencies often surpass what is observed when micturition is initiated. The voiding reflex is rarely triggered when basal pressures are low and constant. Instead, as the bladder expands and basal pressure increases in an exponential fashion, the likelihood of micturition increases rapidly. This suggests that elevated and sustained wall tension, likely incorporating the stretching of passive visco-elastic elements in the bladder wall is an integral part of initiating micturition (29, 30). The voiding reflex may be triggered at a certain volume to prevent over-stretching of the bladder, similar to the peristaltic reflex in response to intestinal over-distention (31). Further studies altering active and passive components of wall tension, although monitoring afferent activity are likely to elucidate how these mechanisms interact during filling and voiding.

### Perspectives and Significance

Voiding of urine involves a complex interplay between biophysical processes occurring in the urinary bladder, sensory information relayed to the CNS, and signal processing within the CNS. When the normal functioning of the lower urinary tract is disrupted, it leads to substantial life complications, such as urinary incontinence, that can be quite disruptive on afflicted individuals. Understanding the normal functioning of the lower urinary tract is an essential element toward a further understanding of how to treat dysfunctions in this system. In our present work, we have determined that, within a broad range of urine production/bladder filling rates, void volumes are maintained quite constant. This constancy in void volumes across a broad range of bladder filling rates occurs despite a heightened input of sensory information into the CNS from the lower urinary tract when the bladder is filled rapidly. We suggest that, in vivo in normal physiological situations, voiding function is not greatly impacted by bladder filling rate, despite heightened sensory outflow from the lower urinary tract (LUT) at faster filling rates. For pathological conditions where heightened sensory outflow from the LUT is thought to exacerbate voiding dysfunction, such as bladder inflammation, stress-associated bladder overactivity, or interstitial cystitis, alterations in bladder filling rate are not likely to contribute to observed voiding dysfunction. Our findings also point to other factors that determine the voiding threshold in vivo, such as arousal state. These intriguing possibilities warrant further investigations.

## GRANTS

This work was supported by National Institutes of Health Grants (R01DK125543, to G. M. Herrera and T. J. Heppner).

## DISCLOSURES

G.M.H. is a scientific consultant at MED Associates, Inc. and Living Systems Instrumentation, a division of Catamount Research and Development, Inc., and his wife is a co-owner of these companies. None of the other authors has any conflicts of interest, financial or otherwise, to disclose.

## AUTHOR CONTRIBUTIONS

T.J.H. and G.M.H. conceived and designed research; T.J.H. and G.M.H. performed experiments; T.J.H. and G.M.H. analyzed data; T.J.H., G.W.H., M.T.N., and G.M.H. interpreted results of experiments; T.J.H. and G.M.H. prepared figures; T.J.H. and G.M.H. drafted manuscript; T.J.H., G.W.H., M.T.N., and G.M.H. edited and revised manuscript; T.J.H., G.W.H., M.T.N., and G.M.H. approved final version of manuscript.

## References

[1] de Groat WC. Anatomy of the central neural pathways controlling the lower urinary tract. Eur Urol 34, Suppl 1: 2–5, 1998. doi:10.1159/000052265. 9705544

[2] Fowler CJ, Griffiths D, de Groat WC. The neural control of micturition. Nat Rev Neurosci 9: 453–466, 2008. doi:10.1038/nrn2401. 18490916 PMC2897743

[3] Daly D, Rong W, Chess-Williams R, Chapple C, Grundy D. Bladder afferent sensitivity in wild-type and TRPV1 knockout mice. J Physiol 583: 663–674, 2007. doi:10.1113/jphysiol.2007.139147. 17627983 PMC2277033

[4] Daly DM, Nocchi L, Liaskos M, McKay NG, Chapple C, Grundy D. Age-related changes in afferent pathways and urothelial function in the male mouse bladder. J Physiol 592: 537–549, 2014. doi:10.1113/jphysiol.2013.262634. 24297847 PMC3930438

[5] Heppner TJ, Tykocki NR, Hill-Eubanks D, Nelson MT. Transient contractions of urinary bladder smooth muscle are drivers of afferent nerve activity during filling. J Gen Physiol 147: 323–335, 2016. doi:10.1085/jgp.201511550. 26976828 PMC4810069

[6] Mills KA, West EG, Sellers DJ, Chess-Williams R, McDermott C. Psychological stress induced bladder overactivity in female mice is associated with enhanced afferent nerve activity. Sci Rep 11: 17508, 2021. doi:10.1038/s41598-021-97053-5. 34471159 PMC8410840

[7] Rong W, Spyer KM, Burnstock G. Activation and sensitisation of low and high threshold afferent fibres mediated by P2X receptors in the mouse urinary bladder. J Physiol 541: 591–600, 2002. doi:10.1113/jphysiol.2001.013469. 12042363 PMC2290323

[8] Tykocki NR, Heppner TJ, Dalsgaard T, Bonev AD, Nelson MT. The KV 7 channel activator retigabine suppresses mouse urinary bladder afferent nerve activity without affecting detrusor smooth muscle K^+^ channel currents. J Physiol 597: 935–950, 2019. doi:10.1113/JP277021. 30536555 PMC6355639

[9] Chakrabarty B, Bijos DA, Vahabi B, Clavica F, Kanai AJ, Pickering AE, Fry CH, Drake MJ. Modulation of bladder wall micromotions alters intravesical pressure activity in the isolated bladder. Front Physiol 9: 1937, 2018. doi:10.3389/fphys.2018.01937. 30687132 PMC6335571

[10] Drake MJ, Hedlund P, Harvey IJ, Pandita RK, Andersson KE, Gillespie JI. Partial outlet obstruction enhances modular autonomous activity in the isolated rat bladder. J Urol 170: 276–279, 2003. doi:10.1097/01.ju.0000069722.35137.e0. 12796704

[11] Drake MJ, Kanai A, Bijos DA, Ikeda Y, Zabbarova I, Vahabi B, Fry CH. The potential role of unregulated autonomous bladder micromotions in urinary storage and voiding dysfunction; overactive bladder and detrusor underactivity. BJU Int 119: 22–29, 2017. doi:10.1111/bju.13598. 27444952 PMC5177525

[12] Parsons BA, Drake MJ, Gammie A, Fry CH, Vahabi B. The validation of a functional, isolated pig bladder model for physiological experimentation. Front Pharmacol 3: 52, 2012. doi:10.3389/fphar.2012.00052. 22479248 PMC3315789

[13] Gillespie JI, van Koeveringe GA, de Wachter SG, de Vente J. On the origins of the sensory output from the bladder: the concept of afferent noise. BJU Int 103: 1324–1333, 2009. doi:10.1111/j.1464-410X.2009.08377.x. 19344428

[14] McCarthy CJ, Zabbarova IV, Brumovsky PR, Roppolo JR, Gebhart GF, Kanai AJ. Spontaneous contractions evoke afferent nerve firing in mouse bladders with detrusor overactivity. J Urol 181: 1459–1466, 2009. doi:10.1016/j.juro.2008.10.139. 19157431 PMC2899488

[15] Kanai A, Andersson KE. Bladder afferent signaling: recent findings. J Urol 183: 1288–1295, 2010. doi:10.1016/j.juro.2009.12.060. 20171668 PMC3686308

[16] Dorr W. Cystometry in mice–influence of bladder filling rate and circadian variations in bladder compliance. J Urol 148: 183–187, 1992. doi:10.1016/s0022-5347(17)36549-7. 1613867

[17] Joseph DB. The effect of medium-fill and slow-fill saline cystometry on detrusor pressure in infants and children with myelodysplasia. J Urol 147: 444–446, 1992. doi:10.1016/s0022-5347(17)37265-8. 1732614

[18] Kim AK, Hill WG. Effect of filling rate on cystometric parameters in young and middle aged mice. Bladder (San Franc) 4: e28, 2017. doi:10.14440/bladder.2017.88. 28553656 PMC5443651

[19] Klevmark B. Volume threshold for micturition. Influence of filling rate on sensory and motor bladder function. Scand J Urol Nephrol Suppl 36: 6–10, 2002. doi:10.1080/003655902320765890. 12475010

[20] Bainier C, Mateo M, Felder-Schmittbuhl MP, Mendoza J. Circadian rhythms of hedonic drinking behavior in mice. Neuroscience 349: 229–238, 2017. doi:10.1016/j.neuroscience.2017.03.002. 28286126

[21] Gilbert RM, Sherman IP. Palatability-induced polydipsia: saccharin, sucrose, and water intake in rats, with and without food deprivation. Psychol Rep 27: 319–325, 1970. doi:10.2466/pr0.1970.27.2.319. 5454116

[22] Herrera GM, Pozo MJ, Zvara P, Petkov GV, Bond CT, Adelman JP, Nelson MT. Urinary bladder instability induced by selective suppression of the murine small conductance calcium-activated potassium (SK3) channel. J Physiol 551: 893–903, 2003. doi:10.1113/jphysiol.2003.045914. 12813145 PMC2343290

[23] Herrera GM, Meredith AL. Diurnal variation in urodynamics of rat. PLoS One 5: e12298, 2010. doi:10.1371/journal.pone.0012298. 20808873 PMC2924395

[24] Ruetten H, Wegner KA, Zhang HL, Wang P, Sandhu J, Sandhu S, Mueller B, Wang Z, Macoska J, Peterson RE, Bjorling DE, Ricke WA, Marker PC, Vezina CM. Impact of sex, androgens, and prostate size on C57BL/6J mouse urinary physiology: functional assessment. Am J Physiol Renal Physiol 317: F996–F1009, 2019. doi:10.1152/ajprenal.00270.2019. 31390231 PMC6843040

[25] Hou XH, Hyun M, Taranda J, Huang KW, Todd E, Feng D, Atwater E, Croney D, Zeidel ML, Osten P, Sabatini BL. Central control circuit for context-dependent micturition. Cell 167: 73–86.e12, 2016. doi:10.1016/j.cell.2016.08.073. 27662084 PMC6217838

[26] Keller JA, Chen J, Simpson S, Wang EH, Lilascharoen V, George O, Lim BK, Stowers L. Voluntary urination control by brainstem neurons that relax the urethral sphincter. Nat Neurosci 21: 1229–1238, 2018. doi:10.1038/s41593-018-0204-3. 30104734 PMC6119086

[27] Tish MM, Geerling JC. The brain and the bladder: forebrain control of urinary (in)continence. Front Physiol 11: 658, 2020. doi:10.3389/fphys.2020.00658. 32719609 PMC7349519

[28] Mawla I, Schrepf A, Ichesco E, Harte SE, Klumpp DJ, Griffith JW, Strachan E, Yang CC, Lai H, Andriole G, Magnotta VA, Kreder K, Clauw DJ, Harris RE, Clemens JQ, Landis JR, Mullins C, Rodriguez LV, Mayer EA, Kutch JJ. Natural bladder filling alters resting brain function at multiple spatial scales: a proof-of-concept MAPP Network Neuroimaging Study. Sci Rep 10: 19901, 2020. doi:10.1038/s41598-020-76857-x. 33199816 PMC7669903

[29] Coolsaet BL, van Duyl WA, van Mastrigt R, van der Zwart A. Visco-elastic properties of the bladder wall. Urol Int 30: 16–26, 1975. doi:10.1159/000279953. 1118945

[30] Wagg A, Fry CH. Visco-elastic properties of isolated detrusor smooth muscle. Scand J Urol Nephrol Suppl 201: 12–18, 1999. 10573771

[31] Waterman SA, Costa M. The role of enteric inhibitory motoneurons in peristalsis in the isolated guinea-pig small intestine. J Physiol 477: 459–468, 1994. doi:10.1113/jphysiol.1994.sp020207. 7932234 PMC1155610

